# Research advances on structure and biological functions of integrins

**DOI:** 10.1186/s40064-016-2502-0

**Published:** 2016-07-15

**Authors:** Li Pan, Yuan Zhao, Zhijie Yuan, Guixin Qin

**Affiliations:** Key Laboratory of Animal Production, Product Quality and Security, Ministry of Education, Key Laboratory of Animal Nutrition and Feed Science, Jilin Province, College of Animal Science and Technology, Jilin Agricultural University, Changchun, People’s Republic of China

**Keywords:** Integrin, Structure, Associated proteins, Signal transduction pathway, Biological function, Adhesion

## Abstract

Integrins are an important family of adhesion molecules that were first discovered two decades ago. Integrins are transmembrane heterodimeric glycoprotein receptors consisting of α and β subunits, and are comprised of an extracellular domain, a transmembrane domain, and a cytoplasmic tail. Therein, integrin cytoplasmic domains may associate directly with numerous cytoskeletal proteins and intracellular signaling molecules, which are crucial for modulating fundamental cell processes and functions including cell adhesion, proliferation, migration, and survival. The purpose of this review is to describe the unique structure of each integrin subunit, primary cytoplasmic association proteins, and transduction signaling pathway of integrins, with an emphasis on their biological functions.

## Background

Integrins are heterodimers consisting of two subunits. Hynes discovered there were 18 α and 8 β subunits forming 24 αβ heterodimers by noncovalent bonds (Hynes 2002). An electron microscope result showed that integrins have a globular head and two leg regions (one from α subunits and the other from β subunits) inserted into the plasma membrane, indicating each integrin subunit has an extracellular domain, a transmembrane domain, and a cytoplasmic tail (Srichai and Zent 2010). The α subunits mainly decide the type of ligands, and both α and β subunits are involved in cell signal transduction which are assisted by the contribution of adhesion molecules. The characteristics of integrin function and molecular diversity were initially clarified in 2000 (Zamir et al. 2000).

Based on the unique structure of integrins, including the α- and β-subunits, integrins can bind with extracellular matrix (ECM) proteins such as collagen (CO), laminin (LN), firbronection (FN), vitronectin (VN), and some other cellular receptors (Plow et al. 2000). The discovery of integrins at molecular level occurred in the late 1970s and 1980s, which was followed by further discoveries of integrin adhesion-related proteins, including structural protein members and signaling molecules (Rohrschneider 1980). Among these, the short intracellular cytoplasmic domains of integrins may associate directly with numerous cytoskeletal proteins and intracellular signaling molecules. These associated proteins provide a basis for modulating fundamental cell processes and various biological outcomes including proliferation, migration, cell differentiation, and apoptosis (Schwartz et al. 1995) by regulating signal transduction pathways. In recent years, many researchers have gradually developed a deep understanding of integrins using techniques like gene knockout, overexpression and specific antibodies. Meanwhile, researchers have also realized the crucial roles of integrins and make a greatly improved understanding for their unique structure, biological function, and integrin-mediated signal transduction mechanism in multiple cellular processes.

This review mainly focuses on the thorough understanding of the different subunit structural characteristics, biological functions, and associated proteins in cells.

## Integrin structure and distribution

### Integrin α subunits

The structures of different α subunits are very similar. The extracellular domains contain 7 homologous repeat domains with 30–40 amino acids, and the interval between these sequences has 20–30 amino acids. Extracellular domains also contain a ‘metal-ion-dependent adhesive site’ (MIDAS) that can bind divalent metal cations (Mg^2+^ or Ca^2+^) and is important in ligand binding. The transmembrane domains of integrins are single-spanning structures with 5 common amino acid sequences, ‘GFFKR’, its specific function is regulating integrin affinity by mediating an alpha–beta subunit cytoplasmic tail interaction. Cytoplasm domains of α subunits are generally short.

**Fig. 1:**
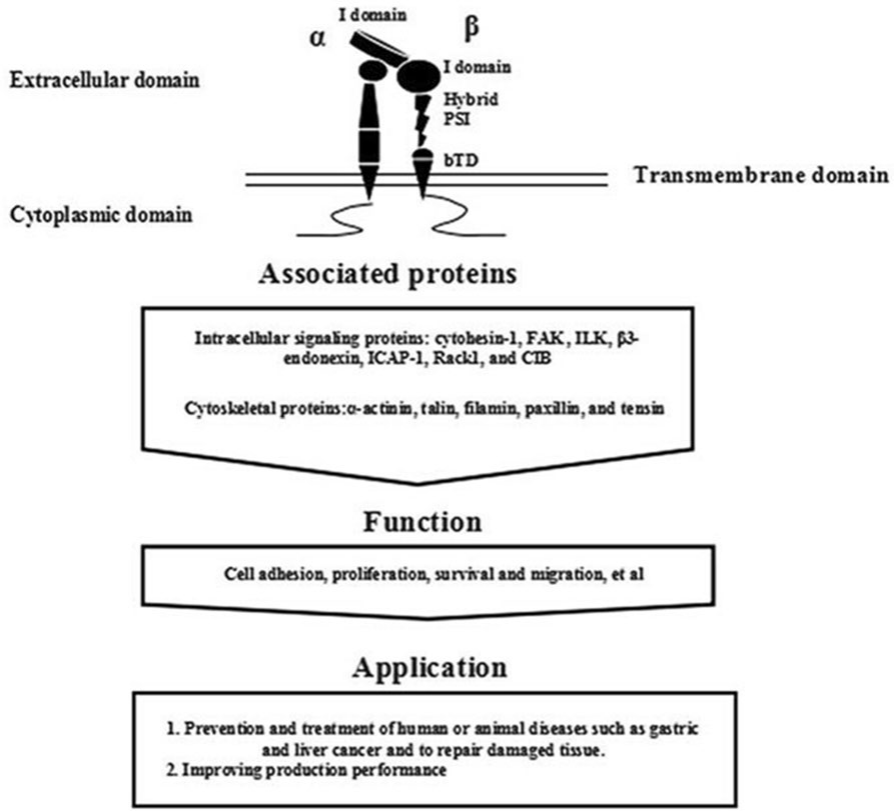
Structure, primary cytoplasmic association proteins and biological functions of integrins

At least 18 α subunits have been found, including α1–α11, αD, αE, αL, αM, αV, αX, and αIIb. To date, their molecular structures have been studied using X-rays, nuclear magnetic resonance, electron microscopy, and three-dimensional ultrasonography. The components of extracellular domains include I-domain, β-Propeller, Thigh, Calf-1, and Calf-2 (Fig. 1). Nine different α subunits (α1, α2, α10, α11, αD, αL, αE, αM, αX) contain the I-domain structure, which is crucial for ligand binding sites. Several other α subunits (α3, α4, α5, α6, α7, α8, α9, αV, αIIb) contain no I-domain but constitute the ligand binding sites by β-Propeller. This article describes the characteristics of α subunit structures and tissue distributions in detail as shown in Table 1.

**Table 1:** Structural characteristics and tissue distributions of α subunits

Integrin subunits	Molecular weight (kDa)	Heterodimeric type	Structural characteristics	Tissue distributions	References
α1	210	α1β1	Has I-domain structure and heavily N-glycosylated when compared with other α chains	Embryo, liver, muscle, several inflammation tissues and epithelial cells	Isacke and Horton (2000)
α2	165	α2β1	Has I-domain structure and three cation binding sites	Epiderm in proliferation basal layer	Isacke and Horton (2000), Teige et al. (2010)
α3	130, 25	α3β1	No I-domain structure although has seven homologous repeated domains and most membrane proximal domains of α3, have divalent cation binding sites	Histological abnormalities of kidney, lungs, small skin blisters and glomerulus	Isacke and Horton (2000), Romanska et al. (2013)
α4	150	α4β1, α4β7	No I-domain structure, otherwise extracellular portions of α4 have three EF-hand loop-like domains for divalent cations binding	Placenta and heart during embryogenesis, also in ladder smooth muscle cells	Isacke and Horton (2000), Luo et al. (2013)
α5	135, 25	α5β1	No I-domain structure, yet has five potential divalent cation binding sites	Embryo, vascellum, wound healing tissues and epithelial cells	Isacke and Horton (2000), Tartaglia et al. (2013)
α6	120, 30	α6β1, α6β4	Structure is most homologous to integrin α3	Platelet, basal surface of most epithelial cells, schwann cells, keratinocytes, prostate cancer cells and endothelial cells	Isacke and Horton (2000), Mercurio et al. (2001), Wilschut et al. (2011), Stewart and O’Connor (2015), Berg et al. (2016)
α7	100, 30	α7β1	No I-domain structure, otherwise proteolytically cleaved	Skeletal muscles, smooth muscles, cardiac muscle and nervous system	(Isacke and Horton 2000)
α8		α8β1	No I-domain structure, yet proteolytically cleaved	Smooth muscles, kidney and epithelial cells	Isacke and Horton (2000), Benoit et al. (2009)
α9		α9β1	No I-domain structure, still post-translationally cleaved	Intestinal epithelia, skin, muscles and liver	Isacke and Horton (2000), Hynes (2002)
α10	160	α10β1	Structure is most homologous to integrin α1 and α2	Heart and skeletal muscles	Hynes (2002), Isacke and Horton (2000)
α11		α11β1	The longest integrin α chain with 1166 amino acids and has I-domain structure, however has no GFFKR sequence	Adult uterus, heart and skeletal muscles	Zhang et al. (2002), Velling et al. (1999)
αv	125, 25	αvβ1, αvβ3, αvβ5, αvβ6, αvβ8	No I-domain structure, otherwise proteolytically cleaved	Neural crest cells, muscles, glial cells, epithelia, osteoclasts, and blood vessels during development or angiogenesis	Delannet et al. (1994), Breuss et al. (1995), Drake et al. (1995), Isacke and Horton (2000), Kaneko et al. (2014)
αIIb	125, 22	αIIbβ3	No I-domain structure, yet proteolytically cleaved	Human blood platelets and macrophagocyte	Isacke and Horton (2000), Ley et al. (2016)
αD	150	αDβ2	Has I-domain structure	Tissue macrophages such as spleen and peripheral blood leucocytes	Isacke and Horton (2000)
αL	180	αLβ2	Has I-domain structure and an imperfect MIDAS, with seven repetitive domains in extracellular domains	Leukocyte receptors	Shimaoka et al. (2002), Springer and Sen (2016)
αM	170	αMβ2	Has I-domain structure although not proteolytically cleaved, with five exposed loops surrounding MIDAS	Leukocyte receptors	Hee et al. (2007)
αX	150	αXβ2	Has I-domain structure yet not proteolytically cleaved, with five exposed loops surrounding MIDAS	Leukocyte receptors	Isacke and Horton (2000)
αE	150, 25	αLβ7	Has I-domain structure even proteolytically cleaved	Leukocyte receptors	Isacke and Horton (2000)

### Integrin β subunits

Integrin β subunits have an I-like domain similar to the I-domain in α subunits which is crucial for ligand binding. Other components include a plexin/semaphorin/integrin (PSI) domain, a hybrid domain, four epidermal growth factor (EGF) repeats, and a membrane proximal b tail domain (bTD), shown in Fig. 1. The β subunits also contain a large extracellular domain, a single-spanning transmembrane domain, and a short cytoplasmic tail (except for β4). The cytoplasmic domains lack catalytic activity themselves and are comprised of 60 amino acids (except for β4, which contains 1000 amino acids) (Hogervorst et al. 1990). Its cytoplasmic domains typically have two NP × Y sequences that provide binding sites to many proteins with phosphotyrosine-binding (PTB) domains (Bouaouina et al. 2008) and participate in cellular signal transduction by linking with cytoplasmic signal molecules (Gilcrease 2007). The super-family of integrin β can be divided into β1–β8 and their structural characteristics and tissue distributions are described in Table 2.

**Table 2:** Structural characteristics and tissue distributions of β subunits

Integrin subunits	Molecular weight (kDa)	Heterodimeric type	Structural characteristics	Tissue distributions	References
β1	115	α1β1, α2β1, α3β1, α4β1, α5β1, α6β1, α7β1, α8β1, α9β1, α10β1, α11β1, αvβ1	Has 56 residues in four repeat regions and internally disulphide bounded	Widely distributed	Isacke and Horton (2000)
β2	95	αDβ2, αLβ2, αMβ2, αXβ2	Cytoplasmic tail contains eight potential phosphorylation sites	Leucocytes	Isacke and Horton (2000), Takada et al. (2007)
β3	105	αvβ3, αIIbβ3	Its Tyr 773 is potentially phosphorylated	Platelets and macrophages	Coppolino and Dedhar (2000), Isacke and Horton (2000), Mor-Cohen (2016)
β4	220	α6β4	Contains a large cytoplasmic domain approximately 1000 amino acids	Epithelial cells	Mercurio et al. (2001)
β5	100	αvβ5		Neural crest cells, blood vessels and tumors	Memmo and McKeown-Longo (1998), Hu et al. (2014)
β6	105	αvβ6	Has a small cytoplasmic extension with unique 11 amino acids	Epithelial cells	Bandyopadhyay and Raghavan (2009)
β7	110	α4β7, αEβ7	Has two NPX(Y/F) motifs for potential tyrosine kinase binding	NK cells, B cells, eosinophils, intraepithelial cells, lymphocytes and peripheral cells	Schippers et al. (2012)
β8	95	αvβ8	No interact with cytoskeleton	Kidney, placenta, uterus, ovary and transformed cell lines	Isacke and Horton (2000)

## Integrin-associated proteins

Integrin cytoplasmic domains associate directly with numerous cytoskeletal proteins and intracellular signaling molecules to modulate fundamental cell processes, as is shown in Fig. 1. Both α and β chains can participate in ligand binding specificity, but β chains alone seem to define cytoskeletal interactions.

The ability of integrin cytoplasmic domains may associate directly with several cytoskeletal proteins including α-actinin, talin, filamin, paxillin, and tensin (Reszka et al. 1992; Otey et al. 1993; Lyman et al. 1997; Geiger et al. 2001). Their binding sites and functions to integrins were summarized in Table 3.

**Table 3:** Integrin-associated proteins (cytoskeletal proteins)

Cytoskeletal proteins	Binding sites to integrins	Functions	Roles in diseases	References
α-actinin	Central repeat of α-actinin can bind to integrin β1 and activated β2 Membrane-spanning region can bind to integrin β3 Cytoplasmic domain position can bind to integrin αIIb	Enhancing signaling from matrix adhesion sites and stimulating integrin-mediated cell-to-matrix adhesion	Non-muscle α-actinins playing roles in the development and progression of cancer, such as metastatic breast, colorectal, pancreatic and ovarian cancer etc	Pavalk and LaRoche (1993), Vinogradova et al. (2002), Kikuchi et al. (2008), Barbolina et al. (2008)
Talin	Containing an atypical four point one protein, ezrin, radixin and moesin (FERM) domain that binding to integrin cytoplasmic tails	Important for transducing signals, actin network organization, focal adhesion composition, and integrin activation	Over expression leading the progression to metastatic disease such as prostate cancer	Desiniotis and Kyprianou (2011), Kim et al. (2011), Goult et al. (2013), Das et al. (2014), Tan et al. (2015)
Filamin	Binding to integrin β tails	Important in integrin signaling transduction and the reorganization of the actin cytoskeleton	Mutation causing congenital anomalies and epileptic seizures	Feng and Walsh (2004), Robertson (2005), Robertson et al. (2006), Kim et al. (2010)
Paxillin	Binding to the membrane proximal region of the integrin β1	Acting as a crucial intermediary in the transduction of signals generated by cell adhesion through integrins	Has an association between Paxillin gene expression and invasive tumor behavior, including lung cancer and breast carcinoma etc	Schaller et al. (1995), Tanaka et al. (1996), Salgia et al. (1999), Madan et al. (2006)
Tensin	Containing a PTB domain	Involved in integrin-mediated focal adhesions	Negativity producing unfavorable prognosis in terms of overall survival in breast cancer	Lo et al. (1994), Yang et al. (2016)

In addition, integrin cytoplasmic domains may also interact directly with several intracellular signaling proteins such as cytohesin-1 (Kolanus et al. 1996), focal adhesion kinase (FAK) (Schaller et al. 1995), integrin-linked kinase (ILK) (Hannigan et al. 1996), β_3_-endonexin (Shattil et al. 1995), cytoplasmic domain associated protein-1 (ICAP-1) (Chang et al. 1997), receptor for activated protein kinase C (Rack1) (Liliental and Chang 1998), and calcium- and integrin-binding protein (CIB) (Naik et al. 1997) (Table 4).

**Table 4:** Integrin-associated proteins (intracellular signaling proteins)

Intracellular signaling proteins	Binding sites to integrins	Functions	Roles in diseases	References
Cytohesin-1	Sec7 domain binds to cytoplasmic tail of integrin β2	Affecting the PI3 K-dependent activation of integrin β2	Regulating human polymorphonuclear neutrophil	Nagel et al. (1998), Azreqa and Bourgoina (2011)
FAK	Directly binding to integrin β1 tail	Playing an essential role in integrin-stimulated signaling mechanism	Important for tumor progression in cancer	Sun et al. (2014)
ILK	C-terminus of ILK binding to the cytoplasmic tails of integrin β1 and β3	Regulating actin cytoskeleton by interacting with various actin-binding actin regulatory proteins and mediating the integrin-dependent signaling	Playing an important function to upregulate several types of cancers, as leukemia	Persad and Dedhar (2003), Böttcher et al. (2009)
β_3_-endonexin	Binding to integrin β3 cytoplasmic tail (Asn-IIe-Thr-Tyr (NITY) motif)	Increasing integrins affinity for ligand	Playing roles in proliferative disease, for example atherosclerosis.	Hannigan et al. (1996)
ICAP-1	C-terminal region containing a PTB domain that providing a binding site for integrin β1	Acting as a messenger that transmits information to the cellular nucleus for controlling gene expression and cell proliferation in a β1-independent manner	Important for body development and pathogenesis	Bouvard et al. (2006), Faurobert et al. (2012)
Rack1	Interacting with the cytoplasmic tails of integrin β1, β2, and β5	Important in the control of integrin-dependent PKC associated signaling cascades	Serving as a scaffold protein in promoting angiogenesis	Liliental and Chang (1998), Li et al. (2000)
CIB	Interacting with integrin	Main function still needing to be tested in a cellular environment		Naik et al. (1997)

## Biological functions and related signaling pathways

Integrins are responsible for sensing many aspects of the cellular microenvironment, including the composition and structure of the ECM and some biochemical signals generated by growth factor or cytokine stimulation. Integrins transmit bidirectional signaling across the plasma membrane by coupling extracellular conformational changes via the unclasping and separation of α and β transmembrane and cytoplasmic domains (Luo and Springer 2006). Inside-out signals regulate integrin affinity for adhesive ligands, outside-in signals depend on ligands that regulate cellular responses to adhesion (Ginsberg et al. 2005). Integrins have no intrinsic catalytic activities, and they transduce intracellular signals via adaptor proteins. Integration of these complex signals contributes to mediate cell biological processes (Parsons et al. 2010).

### Integrins in cell adhesion

Integrin-mediated cell adhesion to extracellular matrix components is essential for the organization, maintenance, and repair of numerous tissues (De and Georges-Labouesse 2000). The cell adhesion process is complex and has a series of steps (Friedl and Wolf 2003), including binding to the extracellular matrix, receptor clustering, and the recruitment of cytoskeletal elements. Integrin-mediated cell adhesion occurs via focal adhesions involving the signaling pathway through ILK (serving as a multifunctional adaptor protein that links focal adhesion to the actin cytoskeleton (Hannigan et al. 2005), FAK, phospholipase C (PLC), and the activation of Pho family proteins. Therein, FAK modulates integrin activity (Lawson et al. 2012) and increases tyrosine phosphorylation in response to integrin activation depending on an intact integrin β cytoplasmic tail (Burridge et al. 1992). The Pho family proteins are important as well. Even the exact relationships between GTPase and integrin mediated-signal pathway are not clear, the integrin-dependent regulation of intracellular PH can occur by Pho GTPase, which has necessary effects on cell spreading and cell adhesion (Tominaga and Barber 1998). The signaling molecules involved in integrin-mediated adhesion are the upstream pathways that mediate other cell functions. Therefore, it is easy to see the link between cell adhesion and other integrin-mediated biological functions such as cell proliferation, survival, and migration. This may explain why integrin α5β1, after binding with FN and intracellular cytoskeletal components located in partial adhesion sites, can induce a series of signal transductions affecting cell motility and migration (Su et al. 2005). Kiwanuka et al. (2013) also indicated that α5β1, αVβ1, and αVβ6 integrin formed adhesions to provide points of traction for cell translocation during keratinocyte migration. Therefore, cell adhesion is the precondition of integrin-mediated biological functions.

### Integrins in cell proliferation

Proliferation of mammalian cells is regulated by various environmental factors, primarily adhesion to ECM. Integrin-mediated adhesion and soluble factors are crucial to cell proliferation, the loss of cell adhesion leads to cell invasion and apoptosis (Blandin et al. 2016). A related study showed that integrins could be an indispensable player during intestinal tumorigenesis and serve as functional platforms to coordinate intestinal stem cell (ISC) maintenance, differentiation, and proliferation in response to environmental factors (Lin et al. 2013). The α2 and α3 subunits displayed an expression spatial gradient in the crypt and were implicated as cell growth patterns and phenotype modulators required for the process of intestinal epithelial cell differentiation (Zhang et al. 2003). Integrins also interact with growth factor receptors and other factors to regulate cell proliferation. Integrins and growth factor receptors can regulate G1 phase cyclins and related kinases that determine the cell cycle via various cytoplasmic signaling pathways (Moreno-Layseca and Streuli 2013; Eberwein et al. 2015).

There are many indications that not all integrin-mediated cell cycle signaling is the same. Most integrins activate FAK, extracellular regulated kinase (ERK), mitogen-activated protein kinases (MAPKs) and Rho family GTPases on rigid ECMs (Luo et al. 2013; Naci and Aoudjit 2014). However, integrin αvβ3 is selectively associated with enhanced signaling by RTK receptors. It can also activate several other pathways including calcium entry into cells (Schwartz and Denninghoff 1994), NF-ΚB (Scatena et al. 1998), and possibly some others. In addition, some integrins cannot induce similar effects despite their similar abilities at promoting cell adhesion and cytoskeletal organization. Integrins αvβ3, α5β1, and α1β1 interact with caveolin to stimulate Shc phosphorylation and possibly other factors to promote DNA synthesis (Wary et al. 1996). Integrins αvβ3 and α5β1 also activate PI3 K, which are phoaphatidylinositol lipids that modify enzymes implicated as mediators of integrin-mediated cytoskeletal changes and play an important role in cell migration (Cary et al. 1999).

### Integrins in cell survival and migration

Cell migration is also vital to various biological phenomena. It is involved in not only normal but also pathological events. For example, cell migration is essential in homeostatic processes such as repairing injured tissues and body immune responses in adults (Steffensen et al. 2001). Cell adhesion receptors are essential for cell migration, and many belong to integrins (Liddington and Bankston 2000). A related report showed that integrin α4β7 had a high expression in mast leukocytes in mucosal inflammation, which promoted the migration of precursor cells to the intestinal tract. Meighan revealed that integrins expressions were up-regulated in migratory cells, and their activities were linked to cellular physiological differentiation (Meighan and Schwarzbauer 2008). During cell migration, integrins must have been recycled or synthesized.

Cell migration involves the localized activation of Rac for the directed protrusion of the cellular membrane only at the leading edges through both the ILK- and FAK-mediated pathways. The intracellular pH and calcium fluxes by integrins also affect cell migration (Schwartz et al. 1989; Marks et al. 1991).

Integrins also play a crucial role in cell survival and protect anchored cells against serum starvation-induced apoptosis. When epithelial and endothelial cell matrix attachment is disrupted, it induces cell apoptosis. Integrin-mediated cell survival is promoted by signaling through the PI3K-AKT, AKT, and ERK pathways (Naci and Aoudjit 2014). If cells are displaced or begin to migrate in an inappropriate environment, they will lose integrin-mediated survival signals (Gilcrease 2007). Signaling through AKT mediates cell survival in adherent epithelial cells by phosphorylating and sequestering BAD, which is a pro-apoptotic Bcl-2 family protein (Cory and Adams 2002). The signaling through the PI3 K-AKT pathway results in the phosphorylation of Bax, which is also a pro-apoptotic Bcl-2 family protein (Gilmore et al. 2000). Integrin-mediated signaling through the ERK pathway down-regulated the pro-apoptotic protein Bim (Reginato et al. 2003).

## Conclusions and future prospects

Integrins have gradually become a research hotspot in cell biology, physiology, genetics, and pathology. Expression levels of integrins in cell membranes not only affect cell morphology, proliferation, differentiation, migration, and some macromolecular syntheses but also important in maintaining organization and structural integrity. According to the specific distribution and function of different integrin subunits, immense researchers have applied the unique structural and biological functions of integrins to study the prevention and treatment of human or animal diseases such as gastric cancer, liver cancer, and damaged tissues, et al. Additionally, integrins transmit bidirectional signaling to exert their biological functions, which plays an important role in cellular processes.

However, many questions remain to be elucidated, such as what the exact regulatory mechanisms are and how to determine integrin-mediated cell proliferation, migration, or survival in different cell or tissue types. Therefore, a better understanding of integrin characteristics and influences on human or animal functioning protein may provide a theoretical basis for clarifying the molecular mechanism of metastasis and solving these problems. The study of integrin-mediated signal transduction will also be an important area of research in the future.
